# Determining clinical significance independently from statistical significance? Implications for practice

**DOI:** 10.1186/1748-7161-7-S1-O34

**Published:** 2012-01-27

**Authors:** S Schreiber, EC Parent, DM Hedden

**Affiliations:** 1 University of Alberta, Faculty of Rehabilitation Medicine, Edmonton, Canada; 2 University of Alberta/Alberta Health Services, Edmonton, Canada

## Objective

To present two proposed methods for determining clinically significant effects and describe each by using the same example from the scoliosis literature where statistical significance was not obtained.

## Background

In science, statistics are universally used for making an inference about a population from sample data. The purpose of statistical inference is to determine if a proposed null hypothesis can be rejected, by comparing the probability of an observation to occur under the null hypothesis (p-value) to a chosen alpha level of confidence. The null hypothesis is rejected if the p-value is smaller than alpha. The current focus on null hypothesis statistical significance testing in published work perpetuates confusion between statistical significance and clinical importance. Clinically, there are shortfalls to relying only on statistical inference.

## Materials and methods

A review of the Pubmed literature revealed several methods for assessing the significance of a clinical effect [1][2]. Two methods will be presented because they are easy to calculate and all variables needed for calculation were available from the scoliosis literature: Half standard deviation rule of thumb and a method combining cut-off points and reliable change index (RCI). Both methods are presented using an example from the scoliosis literature on the effect of exercises where statistical significance was not obtained.

## Results

The proposed methods, although mathematically different, are more similar than different in terms of the conclusions they produce.

## Conclusions

A combination of statistical and clinical significance determination methods to drawn statistically and clinically relevant inferences should be used when reporting clinical study results.

## References

[B1] Wise EA Statistical significance testing and clinical effectiveness studies Psychotherapy (chic) 2011 48 3 225 8 10.1037/a0022701 21875236

[B2] Chapman JR Norvell DC Hermsmeyer JT Bransford RJ Devine J McGirt MJ Lee MJ Evaluating common outcomes for measuring treatment succes for chronic low back pain Spine 2011 36 21 S54 68 21952190 10.1097/BRS.0b013e31822ef74d

